# Supplementary material for: Plant-derived angiogenin fusion protein’s cytoprotective effect on trabecular meshwork damage induced by Benzalkonium chloride in mice
Source: PeerJ. 2020 May 22;8:e9084. doi: 10.7717/peerj.9084 (PMC7247531; doi:10.7717/peerj.9084)
Supplement: Supplemental Information 1 [file peerj-08-9084-s001.pdf]

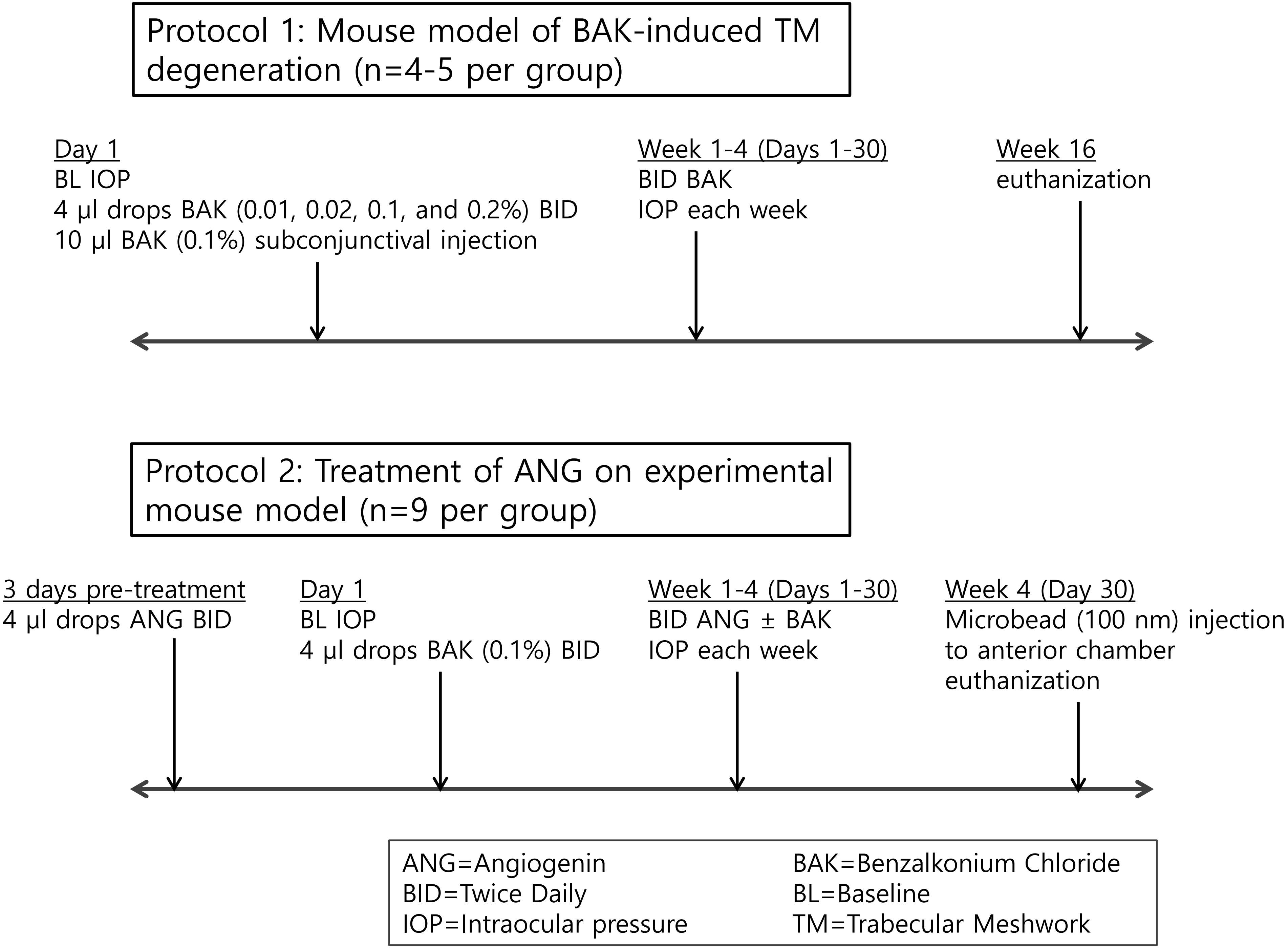
Protocol 1: Mouse model of BAK-induced TM degeneration (n=4-5 per group)
Day 1
BL IOP
4 µl drops BAK (0.01, 0.02, 0.1, and 0.2%) BID
10 µl BAK (0.1%) subconjunctival injection
Week 1-4 (Days 1-30)
BID BAK
IOP each week
Week 16
euthanization
Protocol 2: Treatment of ANG on experimental mouse model (n=9 per group)
3 days pre-treatment
4 µl drops ANG BID
Day 1
BL IOP
4 µl drops BAK (0.1%) BID
Week 1-4 (Days 1-30)
BID ANG ± BAK
IOP each week
Week 4 (Day 30)
Microbead (100 nm) injection to anterior chamber
euthanization
ANG=Angiogenin
BID=Twice Daily
IOP=Intraocular pressure
BAK=Benzalkonium Chloride
BL=Baseline
TM=Trabecular Meshwork
