# Supplementary figures and images for: Plant-derived angiogenin fusion protein’s cytoprotective effect on trabecular meshwork damage induced by Benzalkonium chloride in mice
Source: PeerJ. 2020 May 22;8:e9084. doi: 10.7717/peerj.9084 (PMC7247531; doi:10.7717/peerj.9084)

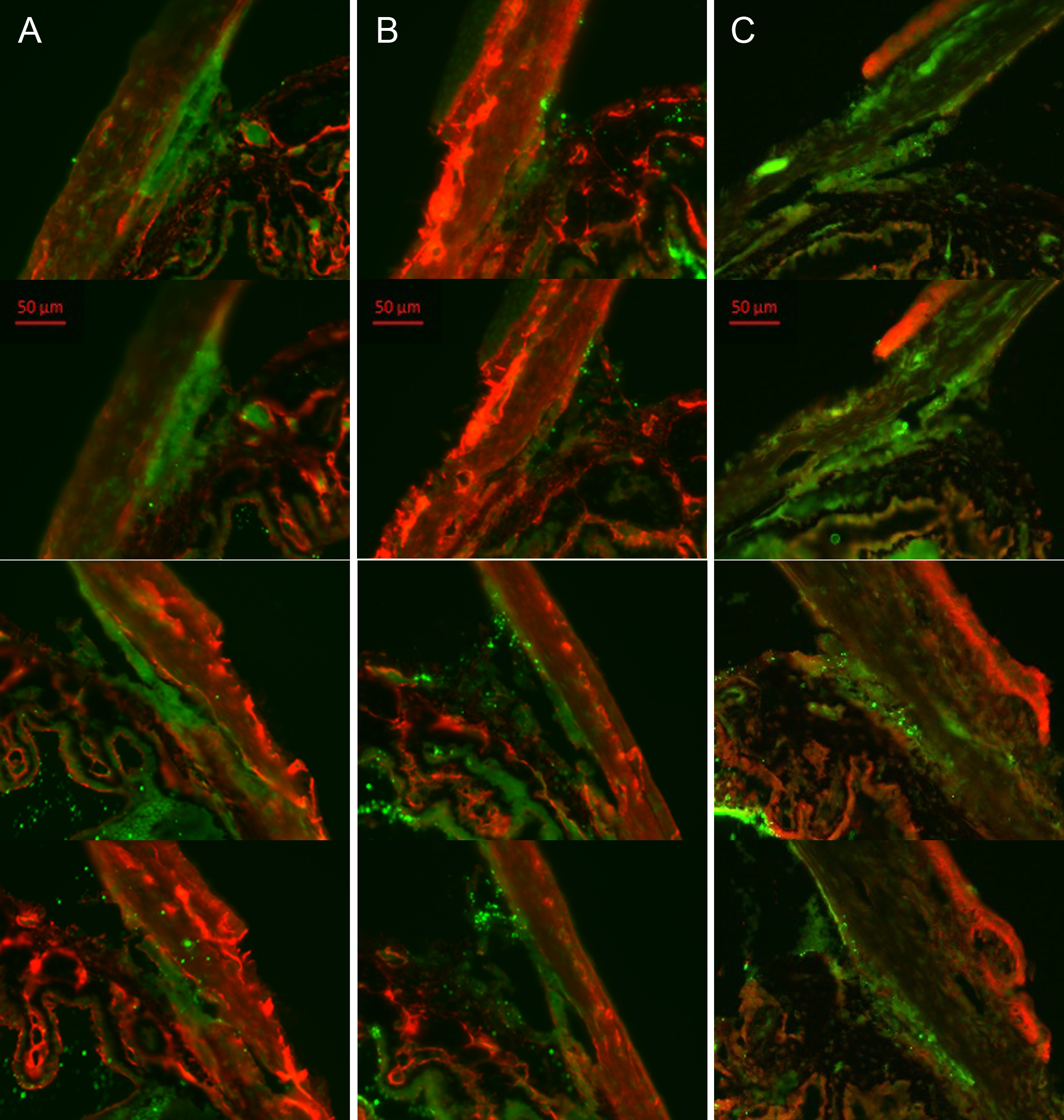

Supplement: Supplemental Information 2 — The distribution of green fluorescent microbeads of aqueous outflow tissues was sparse in continuous parallel sections of all 3 single BAK-treated mouse models (A, B, C). [file peerj-08-9084-s002.png]
